# 2598. Activity of Aztreonam-Avibactam and Other β-Lactamase Inhibitor Combinations against Gram-negative Bacteria Isolated from Patients Hospitalized with Pneumonia in US Medical Centers (2020–2022)

**DOI:** 10.1093/ofid/ofad500.2213

**Published:** 2023-11-27

**Authors:** Helio S Sader, Rodrigo E Mendes, Leonard R Duncan, Timothy Doyle, Mariana Castanheira

**Affiliations:** JMI Laboratories, North Liberty, Iowa; JMI Laboratories, North Liberty, Iowa; JMI Laboratories, North Liberty, Iowa; JMI Laboratories, North Liberty, Iowa; JMI Laboratories, North Liberty, Iowa

## Abstract

**Background:**

Resistance to recently approved β-lactamase inhibitor combinations, such as ceftazidime-avibactam (CAZ-AVI) and meropenem-vaborbactam (MEM-VAB), appears to be increasing among carbapenem-resistant Enterobacterales (CRE) in some US hospitals. We evaluated the frequency and antimicrobial susceptibility of Gram-negative bacteria (GNB) causing pneumonia in US hospitals.

**Figure:**
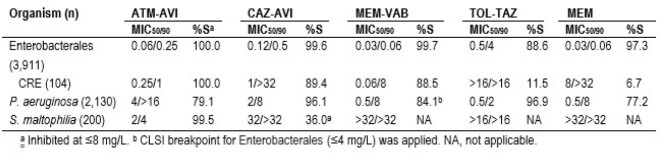

**Methods:**

Bacterial isolates were consecutively collected (1/patient) from patients hospitalized with pneumonia and the susceptibility of GNBs (3,911 Enterobacterales [ENT] and 2,753 non-fermenters) was evaluated by broth microdilution. Isolates were collected in 69 medical centers in 2020–2022. Susceptibility testing was performed by CLSI broth microdilution in a monitoring laboratory. Aztreonam-avibactam (ATM-AVI) was tested with AVI at fixed 4 mg/L and a pharmacokinetic/pharmacodynamic susceptible (S) breakpoint of ≤ 8 mg/L was applied for comparison. CRE isolates were screened for carbapenemases (CPE) by whole genome sequencing.

**Results:**

GNB represented 71.1% of organisms. The most common GNB species were *P. aeruginosa* (22.4% of organisms), *K. pneumoniae* (8.6%), *E. coli* (6.6%), *S. marcescens* (6.2%), *S. maltophilia* (4.9%), and *E. cloacae* complex (4.8%). ATM-AVI inhibited 100.0% of ENT at ≤ 8 mg/L and 99.9% at ≤ 4 mg/L and showed potent activity against CRE (MIC_50/90_, 0.25/1 mg/L; Table). CAZ-AVI and MEM-VAB were active against 89.4% and 88.5% of CREs, respectively. ATM-AVI retained activity against ENT non-S to CAZ-AVI and/or MEM-VAB (n=19; MIC_50/90_, 0.25/4 mg/L). The most common CPEs were KPC (69.2% of CREs), NDM (9.6%), and SME (4.8%). A CPE gene was not observed in 16.3% of CREs. CAZ-AVI and MEM-VAB were highly active against KPC and SME producers but showed limited activity against MBL producers. The most active comparators against CRE were tigecycline (95.2%S), amikacin (73.1%S), and gentamicin (60.6%S). Among *P. aeruginosa*, 79.1% were inhibited at ≤8 mg/L of ATM-AVI, 77.2% were MEM-S, and 77.2% were piperacillin-tazobactam-S. ATM-AVI was highly active against *S. maltophilia*, inhibiting 99.5% of isolates at ≤ 8 mg/L.

**Conclusion:**

ATM-AVI demonstrated potent *in vitro* activity against the GNB most isolated from patients with pneumonia in US hospitals.

**Disclosures:**

**Helio S. Sader, MD, PhD, FIDSA**, AbbVie: Grant/Research Support|Basilea: Grant/Research Support|Cipla: Grant/Research Support|Paratek: Grant/Research Support|Pfizer: Grant/Research Support|Shionogi: Grant/Research Support **Rodrigo E. Mendes, PhD**, AbbVie: Grant/Research Support|Basilea: Grant/Research Support|Cipla: Grant/Research Support|Entasis: Grant/Research Support|GSK: Grant/Research Support|Paratek: Grant/Research Support|Pfizer: Grant/Research Support|Shionogi: Grant/Research Support **Leonard R. Duncan, PhD**, AbbVie: Grant/Research Support|Basilea: Grant/Research Support|CorMedix: Grant/Research Support|Melinta: Grant/Research Support|Pfizer: Grant/Research Support **Timothy Doyle, MS**, AbbVie: Grant/Research Support **Mariana Castanheira, PhD**, AbbVie: Grant/Research Support|Basilea: Grant/Research Support|bioMerieux: Grant/Research Support|Cipla: Grant/Research Support|CorMedix: Grant/Research Support|Entasis: Grant/Research Support|Melinta: Grant/Research Support|Paratek: Grant/Research Support|Pfizer: Grant/Research Support|Shionogi: Grant/Research Support

